# Supplementary material for: Midwives speaking out on COVID-19: The international confederation of midwives global survey
Source: PLoS One. 2022 Nov 2;17(11):e0276459. doi: 10.1371/journal.pone.0276459 (PMC9629587; doi:10.1371/journal.pone.0276459)
Supplement: S1 File — (PDF) [file pone.0276459.s001.pdf]

International Confederation of Midwives

Strengthening Midwifery Globally

ICM GLOBAL COVID 19 RESPONSE SURVEY

What is the ICM COVID-19 Study?

The ICM is coordinating a global research study to understand the challenges and concerns of Midwives' professional associations during the COVID-19 pandemic outbreak.
We hope that our research can help build a greater understanding of the important role of midwives' professional associations in supporting midwives and quality maternal and new-born health services for women and their families globally.

This study has been developed by a global team of midwife researchers to understand how maternity and midwifery care has changed since the outbreak of COVID-19. ICM aims to determine the impact and response of Midwives' Associations to the pandemic and to identify the issues that continue to face women and midwives. The research raises awareness of the role of Midwives' associations and will inform policy and practices at local, national, and global levels.

The Participant Information Sheet and Consent Form are attached within your email invitation.

The survey refers to the first three months, or when COVID -19 first arrived in your country. At the end of the survey, there is an opportunity to let us know whether things have changed and in what way.

Completion of this survey confers consent to participate.

Thank you

International Confederation of Midwives

Strengthening Midwifery Globally

ICM GLOBAL COVID 19 RESPONSE SURVEY

Midwives' Association Information

* 1. Name and role of respondent

* 2. Email address of respondent

* 3. Name of the Midwives' Asssociation

* 4. Country

* 5. ICM Region

- ◯ North America and the Caribbean
- ◯ Latin America
- ◯ Francophone Africa
- ◯ Anglophone Africa
- ◯ Northern Europe
- ◯ Central Europe
- ◯ Southern Europe
- ◯ Eastern Mediterranean
- ◯ South East Asia
- ◯ Western Pacific

6. Human Development Index for your country if known

International Confederation of Midwives

Strengthening Midwifery Globally

What were the issues specifically facing midwives from the outset of COVID 19?

* 7. Were Midwives' Associations informed of the dangers of the SARS-COV-2 virus (COVID-19) at the outset?

- ◯ Yes
- ◯ Not informed
- ◯ Not known
- ◯ Other (please specify)

* 8. Where did Midwives' Associations access COVID-19 information? (Click all relevant sources)

- ☐ Employers
- ☐ Media
- ☐ Health Department or Ministry of Health
- ☐ Other Government Departments
- ☐ ICM
- ☐ UN Departments

Other (please specify)

* 9. Was there a national strategy for the provision of Personal Protection Equipment?

- ◯ Yes
- ◯ No
- ◯ Unclear

* 10. Were midwives offered Personal Protection Equipment at the outset of COVID 19 (30th January 2020)?

- ◯ Yes
- ◯ No
- ◯ Unknown

International Confederation of Midwives

Strengthening Midwifery Globally

ICM GLOBAL COVID 19 RESPONSE SURVEY

11. If Yes, who provided Personal Protection Equipment to midwives?

- [ ] Employers
- [ ] Health Department or Ministry of Health
- [ ] Other Government Department
- [ ] Other (please specify)

* 12. Was there a lack of or a shortage of Personal Protection Equipment for midwives?

- ( ) Yes
- ( ) No
- ( ) Unsure

* 13. What Personal Protection Equipment resources were available?

- [ ] Gloves
- [ ] Masks
- [ ] Full Personal Protection Equipment
- [ ] Unsure

Other (please specify)

* 14. Who did receive Personal Protection Equipment?

- [ ] Midwives
- [ ] Nurses
- [ ] Doctors
- [ ] Unsure

Other (please specify)

* 15. Did your Association need to negotiate for the supply of Personal Protection Equipment for midwives?

- ○ Yes
- ○ No

* 16. What did midwives do if there were shortages of Personal Protection Equipment?

- [ ] Make their own
- [ ] Purchase their own
- [ ] Improvise with whatever was available
- [ ] Re-use single use equipment
- [ ] Work without Personal Protection Equipment
- [ ] Not attend work
- [ ] Not known
- [ ] Other (please specify)

* 17. Did midwives receive training to manage infected women?

- ○ Yes
- ○ No
- ○ Unsure

* 18. Were midwives afraid to go to maternity facilities?

- Yes
- No
- Not known

Other (please specify)

* 19. Did midwives receive their salary during the pandemic?

- Yes
- No
- Not on regular basis

* 20. Did midwives and other health professionals collaborate and join forces to respond to COVID-19?

- Yes
- No
- Unsure

International Confederation of Midwives

Strengthening Midwifery Globally

ICM GLOBAL COVID 19 RESPONSE SURVEY

21. Please describe the collaborations or partnerships with other health care professionals.

* 22. Were midwives deployed outside maternity services to care for COVID -19 patients?

- Yes
- No
- Unsure

ICM GLOBAL COVID 19 RESPONSE SURVEY

23. If yes, were the deployed midwives kept safe

- ◯ Yes
- ◯ No

* 24. Were midwives required to undertake nursing roles?

- ◯ Yes
- ◯ No

International Confederation of Midwives
Strengthening Midwifery Globally

ICM GLOBAL COVID 19 RESPONSE SURVEY

* 25. Were these nursing roles different to before the onset of COVID-19?

- ◯ Yes
- ◯ No

* 26. Were there midwife shortages for maternity care?

- ◯ Yes
- ◯ No

* 27. Were midwives required to work longer hours?

- ◯ Yes
- ◯ No

* 28. Were midwives being paid for extra responsibilities?

- Yes
- No

* 29. Were midwives confident that they were sufficiently protected at all times when offering midwifery care during the pandemic?

- [ ] No, not at all
- [ ] Yes, during antenatal care
- [ ] Yes, during labour and birth care
- [ ] yes, during postnatal care
- [ ] yes, during routine visits
- [ ] Yes, all the time

Other (please specify)

* 30. Were midwives advised how to conduct virtual antenatal visits (eg by telehealth)?

- Yes
- No
- Not applicable

Other (please specify)

* 31. Were midwives advised how to conduct virtual postnatal care

- Yes
- No
- Not applicable

Other (please specify)

ICM GLOBAL COVID 19 RESPONSE SURVEY

32. If yes, who provided this advice or training about virtual antenatal or postnatal visits?

- [ ] Midwives Association
- [ ] Department of Health or Ministry of Health
- [ ] Employer
- [ ] WHO
- [ ] UNFPA
- [ ] ICM

Other (please specify)

* 33. Were midwives concerned about any of the following, if they were found to have missed something during virtual visits?

- [ ] Not applicable, the midwives didn't express concern
- [ ] May have broken the law
- [ ] May be subjected legal action
- [ ] May be in breach of their practice standards
- [ ] May be deregistered
- [ ] Other (please specify)

* 34. Did midwives have to pay for their COVID -19 tests?

- [ ] Yes
- [ ] No
- [ ] Not known

* 35. What happened to midwives who became infected with COVID-19?

* 36. Did midwives have to pay for their treatment?

- ( ) Yes
- ( ) No
- ( ) Not known

* 37. Did midwives die due to COVID-19?

- ( ) Yes
- ( ) No
- ( ) Unknown

Other (please specify)

International Confederation of Midwives

Strengthening Midwifery Globally

ICM GLOBAL COVID 19 RESPONSE SURVEY

38. How many midwives died in your country?

39. Why did midwives die?

International Confederation of Midwives

Strengthening Midwifery Globally

ICM GLOBAL COVID 19 RESPONSE SURVEY

What were the issues facing women (that affected the response of midwives)?

* 40. Were women afraid to go to maternity facilities?

- Yes
- No
- Unknown

Other (please specify)

* 41. Has there been a reduction in attendance by women at maternity facilities during the pandemic?

- Yes
- No
- Unknown

Other (please specify)

* 42. Was there a lack of clear information and guidance for women?

- Yes
- No
- Unknown

* 43. Who provided the information about COVID-19 to women?

- [ ] Health Department or Ministry of Health
- [ ] Other Government Departments
- [ ] Midwife Association
- [ ] Social Media platforms
- [ ] Mainstream media radio or television
- [ ] Not clear who provided the information

44. If the information source was unclear, did the women receive or have access to:

- [ ] Text messages
- [ ] Hot lines
- [ ] Radio messages
- [ ] Print material
- [ ] Social media

Other (please specify)

* 45. Did women experience increased levels of obstetric intervention during birth such as:

- [ ] Induction of labour
- [ ] Caesarean

Other (please specify)

* 46. Were babies separated from mothers?

- ( ) Yes
- ( ) No

Other (please specify)

ICM GLOBAL COVID 19 RESPONSE SURVEY

47. If yes, was this due to:

- ◯ Maternity facility policy relating to COVID-19 infection control
- ◯ Suspected COVID-19 infection
- ◯ Confirmed COVID-19 infection
- ◯ Unknown reasons

Other (please specify)

* 48. Were mothers with suspected or confirmed COVID-19 discouraged from breastfeeding?

- ◯ Yes
- ◯ No
- ◯ Unknown

Other (please specify)

* 49. Was there a policy to discourage all new mothers from breastfeeding during the pandemic?

- ◯ Yes
- ◯ No
- ◯ Unknown

Other (please specify)

* 50. Was there conflicting advice for women and midwives around the safety of breastfeeding during the pandemic?

- ◯ Yes
- ◯ No

Other (please specify)

* 51. Were women concerned that they did not have information on how to protect their new-born babies?

- ◯ Yes
- ◯ No
- ◯ Unknown

Other (please specify)

* 52. Did women report that they felt that their rights were denied?

- Yes
- No
- Unknown

Other (please specify)

* 53. Were some maternity facilities closed to become COVID-19 treatment facilities?

- Yes
- No
- Other (please specify)

* 54. Do you think women were more likely to give birth without a skilled attendant?

- Yes
- No
- Not known
- Other (please specify)

* 55. Were women forced to go to a different facility from where they had originally planned to give birth?

- Yes
- No
- Unknown

Other (please specify)

* 56. Were women able to access midwives for:

- All of their care
- Antenatal care
- Labour and birth care
- Postnatal care
- Routine visits
- None of their care

Other (please specify)

* 57. Was there an increased demand for community and home-based care from a midwife?

- Yes
- No
- Other (please specify)

* 58. Were women able to access midwives to provide homebirth care?

- Yes
- No
- Not relevant, we don't have homebirth services in our country.

59. How did women access home birth services?

* 60. Were women denied a support person or partner during labour?

- ◯ Yes
- ◯ No

Other (please specify)

* 61. Did women report an increase in domestic violence?

- ☐ Yes
- ☐ No
- ☐ Not known
- ☐ Other (please specify)

* 62. Did women experience a lack of access to contraception?

- ◯ Yes
- ◯ No
- ◯ Women don't have access to contraception in our country

* 63. Did women experience a lack of access to safe abortion?

- ◯ Yes
- ◯ No
- ◯ Women don't have access to safe abortion in our country.

* 64. Is your Association expecting an increase in unplanned pregnancies?

- ◯ Yes
- ◯ No

ICM GLOBAL COVID 19 RESPONSE SURVEY

How visible was guidance from Governments, Departments of Health or Ministries of Health?

* 65. When did Midwifery Associations receive government, Department of Health or Ministry of Health advice?

- ◯ Immediately
- ◯ Within 1-2 weeks
- ◯ Not at all
- ◯ Other (please specify)

* 66. What was the advice specifically about?

- ☐ Screening for COVID-19
- ☐ Management of COVID-19
- ☐ Other (please specify)

* 67. How was the initial governmental guidance provided?

- [ ] Health Department or Ministry of Health advice
- [ ] Health facility management or employer
- [ ] Text messages
- [ ] Popular Media
- [ ] Social media
- [ ] Website - name the website/s eg WHO

* 68. How were the governmental COVID-19 pandemic updates provided?

- [ ] Health Department or Ministry of Health advice
- [ ] Health facility management or employer
- [ ] Text messages
- [ ] Popular Media
- [ ] Social media
- [ ] Website - name the website/s eg WHO

* 69. Did midwives receive information or updates from other sources?

- [ ] ICM
- [ ] WHO
- [ ] Employer
- [ ] Social media
- [ ] Unspecified text
- [ ] Other (please specify)

* 70. Was the guidance easy to find and accessible?

- ( ) Yes
- ( ) No

* 71. Was the guidance clear and explicit?

- ◯ Yes
- ◯ No

* 72. Was there a government/ Department of Health or Ministry of Health portal for practitioners to have questions answered?

- ◯ Yes
- ◯ No

* 73. Did the Midwives' Association access information from the ICM website?

- ◯ Yes
- ◯ No

Other (please specify)

International Confederation of Midwives

Strengthening Midwifery Globally

ICM GLOBAL COVID 19 RESPONSE SURVEY

What maternity services were affected?

* 74. Were midwives required to provide the following care that was not usual in their countries before COVID-19?

- [ ] Community-based care
- [ ] Home based care
- [ ] Telephone-based antenatal or postnatal care
- [ ] Virtual or internet based
- [ ] Mobile clinics
- [ ] No change
- [ ] Other (please specify)

* 75. Was there an increased demand for the following?

- [ ] Home birth
- [ ] Birth in the community
- [ ] Community-based or home-based antenatal care
- [ ] Community-based or home-based postnatal care
- [ ] On-line consultations
- [ ] None of the above
- [ ] Other (please specify)

* 76. Were maternity units discharging women earlier than usual ?

- ( ) Yes, with follow up care
- ( ) Yes, with NO follow up care
- ( ) No

Other (please specify)

* 77. Were maternity units discharging women earlier than usual after caesarean section?

- ◯ Yes, with follow up care
- ◯ Yes, with NO follow up care
- ◯ No

Other (please specify)

* 78. What services were closed because of Covid-19?

- ☐ Sexual health services
- ☐ Family planning services
- ☐ Well child services
- ☐ None
- ☐ Other (please specify)

* 79. Are these services still closed?

- ◯ Yes
- ◯ No

* 80. What services reduced their hours because of Covid-19?

- ☐ Sexual health services
- ☐ Family planning services
- ☐ Well child services
- ☐ None
- ☐ Other (please specify)

International Confederation of Midwives

Strengthening Midwifery Globally

How was midwifery education affected?

* 81. Were midwifery education programmes closed?

- ○ Yes
- ○ No

* 82. What alternative modes of learning have been provided to students to ensure continuity of midwifery education?

- ☐ Online learning
- ☐ Small group learning
- ☐ None

Other (please specify)

* 83. Were midwifery students expected to provide general nursing care for COVID-19 patients?

- ○ Yes
- ○ No
- ○ Unknown

* 84. Did students have access to practice areas or clinical placements?

- ○ Yes always
- ○ Yes sometimes
- ○ No

* 85. Were there delays in completing their studies to enable them to register as midwife?

- ○ Yes
- ○ No

* 86. What other changes have occurred in midwifery education since the pandemic began?

International Confederation of Midwives

Strengthening Midwifery Globally

ICM GLOBAL COVID 19 RESPONSE SURVEY

How did Midwives' Associations respond to the pandemic?

* 87. Did your Association advocate for the provision of protective equipment?

- ○ Yes
- ○ No

* 88. Did your Association advocate for COVID-19 testing of your midwives?

- ○ Yes
- ○ No

* 89. Did your Association advocate on behalf of midwives for:

- ☐ Community-based care
- ☐ Procurement of Personal Protection Equipment
- ☐ Distribution of Personal Protection Equipment
- ☐ Safer working conditions
- ☐ Preventing midwives being redeployed
- ☐ Preventing maternity service closures
- ☐ Home birth
- ☐ Other (please specify)

* 90. To what extent did your Association advocate for home birth?

- Provided a lot of advocacy for home birth
- Provided some advocacy for home birth
- Provided no advocacy for home birth
- Provided advocacy for community based care
- Home birth is not an option in our country

* 91. Was your Midwives' Association invited to contribute to government policies relating to COVID-19?

- Yes
- No

Other (please specify)

* 92. Was your Midwifery Association represented in a national health planning committee?

- Yes
- No
- Other (please specify)

* 93. Did your Midwives’ Association call for legislative changes?

- Yes
- No

* 94. What was the focus of the call for legislative changes?

- [ ] Midwife autonomy
- [ ] Home birth
- [ ] Community services
- [ ] Prevention of maternity facility closure
- [ ] Better access to public funding e.g. medicare for telehealth?
- [ ] Other (please specify)

* 95. Was your Association supported or challenged by other professions?

- ○ Yes
- ○ No
- ○ If yes, specify the professions.

* 96. If there are other Midwives' Associations or professional Associations that accept midwives in your country, did you collaborate and support each other during the pandemic?

- ○ Yes
- ○ No
- ○ There are no other Associations that accept midwives

* 97. Were Midwives' Associations consulted around the government's COVID-19 policy?

- ○ Yes all the time
- ○ Yes sometimes
- ○ No, not at all

Other (please specify)

International Confederation of Midwives

Strengthening Midwifery Globally

ICM GLOBAL COVID 19 RESPONSE SURVEY

* 98. Who consulted your Association?

- [ ] Health Department or Ministry of Health
- [ ] Government
- [ ] We were not consulted
- [ ] Others (please specify)

* 99. How would you describe the role of your Midwives' Association during the pandemic?

* 100. Would you agree that midwives are better prepared for future COVID-19 outbreaks?

- ( ) Yes
- ( ) No
- ( ) Unknown

* 101. Can you describe how things are different now than they were at the beginning of the pandemic

* 102. What do you imagine the future for Midwifery will look like in your country in 12 months time from now?

* 103. Can you provide examples of midwifery advocacy led by your Midwives’ Association that has led to positive change for women during this current pandemic?

* 104. Are you prepared to provide a brief case study (story) to illustrate the role of your Midwives’ Association?

- Yes
- No

* 105. Would your Association be willing to have a follow-up interview with ICM?

- Yes
- No

106. Contact email for the follow-up interview.
