# Supplementary material for: Midwives speaking out on COVID-19: The international confederation of midwives global survey
Source: PLoS One. 2022 Nov 2;17(11):e0276459. doi: 10.1371/journal.pone.0276459 (PMC9629587; doi:10.1371/journal.pone.0276459)
Supplement: Attachment — Submitted filename: Response to Reveiwers.docx [file pone.0276459.s002.docx]

Associate Professor Donna Hartz

C/- The University of Newcastle

School of Nursing and Midwifery

Gosford Precinct

77 Holden St,

Gosford. NSW 2250

AUSTRALIA

February 28 2022

Dear Editor-In-Chief PLOS ONE, Dr Emily Chenette,

Thank you for the opportunity to respond to the reviewer’s and editor’s feedback and requests. Please find our reviewed study manuscript (both marked and unmarked) titled “**Midwives Speaking Out on COVID-19: The International Confederation of Midwives (ICM) Global Survey”** for your consideration.

As requested, the following tables address each point raised.

| **Editors Comments** | **Response** | |
| --- | --- | --- |
| 1. Please ensure that your manuscript meets PLOS ONE's style requirements, including those for file naming. | This has been reviewed and complied with. | |
| 2. Please provide additional details regarding participant consent. In the ethics statement in the Methods and online submission information, please ensure that you have specified (1) whether consent was informed and (2) what type you obtained (for instance, written or verbal, and if verbal, how it was documented and witnessed). If your study included minors, state whether you obtained consent from parents or guardians. If the need for consent was waived by the ethics committee, please include this information. If you are reporting a retrospective study of medical records or archived samples, please ensure that you have discussed whether all data were fully anonymized before you accessed them and/or whether the IRB or ethics committee waived the requirement for informed consent. If patients provided informed written consent to have data from their medical records used in research, please include this information. | See line L138-143: The Participant information sheet informed the participants the this was voluntary and a response to the survey implied consent  L135-141:“The study design is a descriptive cross-sectional online, web based, open-link survey. An email from the headquarters of ICM in The Hague was sent to every midwife association registered with ICM on the 1^st^ of July 2020 inviting them to participate in the ICM COVID-19 survey. The participant information sheet, consent form and survey link to the survey was included. Participation was on a voluntary basis and a response to the survey implied consent. One response per association in either English, French or Spanish was invited.”  This study:   - did not include minors, - ethics was not applied for consent to be waived, - is not a retrospective study of medical records or samples. | |
| 3. Thank you for stating the following in the Acknowledgments Section of your manuscript:  We also acknowledge the financial support of UNFPA and Johnson and Johnson™.  We note that you have provided funding information that is not currently declared in your Funding Statement. However, funding information should not appear in the Acknowledgments section or other areas of your manuscript. We will only publish funding information present in the Funding Statement section of the online submission form.  Please remove any funding-related text from the manuscript and let us know how you would like to update your Funding Statement. Currently, your Funding Statement reads as follows:  Contracted project. United National Population Fund (UNFPA): [https://www.unfpa.org](https://protect-au.mimecast.com/s/dol9CmO5k3TD31r5CDltvp?domain=unfpa.org) UNFPA played no direct role in the undertaking of this research. A UNFPA representative (Dr Bar-Zeev) was member of the steering committee and due to her unique role in maternal health in UNFPA contributed to the study design and survey tool development | The funding by UNFPA and Johnson and Johnson were included incorrectly and has been deleted.  This paragraph is confirmed for the funding statement.  This was a contracted project by the United Nations Population Fund (UNFPA) through the International Confederation of Midwives (ICM). UNFPA played no direct role in the undertaking of this research. A UNFPA representative was a member of the steering committee. |  |
| 4. In your Data Availability statement, you have not specified where the minimal data set underlying the results described in your manuscript can be found. PLOS defines a study's minimal data set as the underlying data used to reach the conclusions drawn in the manuscript and any additional data required to replicate the reported study findings in their entirety. All PLOS journals require that the minimal data set be made fully available. For more information about our data policy, please see [http://journals.plos.org/plosone/s/data-availability](https://protect-au.mimecast.com/s/SvE0Cnx1l3Tj2lYGtpaLRy?domain=journals.plos.org).  "Upon re-submitting your revised manuscript, please upload your study’s minimal underlying data set as either Supporting Information files or to a stable, public repository and include the relevant URLs, DOIs, or accession numbers within your revised cover letter. For a list of acceptable repositories, please see [http://journals.plos.org/plosone/s/data-availability#loc-recommended-repositories](https://protect-au.mimecast.com/s/05-YCoV1m5cZRl0Xh7nlWI?domain=journals.plos.org). Any potentially identifying patient information must be fully anonymized.  Important: If there are ethical or legal restrictions to sharing your data publicly, please explain these restrictions in detail. Please see our guidelines for more information on what we consider unacceptable restrictions to publicly sharing data: [http://journals.plos.org/plosone/s/data-availability#loc-unacceptable-data-access-restrictions](https://protect-au.mimecast.com/s/KU7ECp81n3TKWQBzFxM0-L?domain=journals.plos.org). Note that it is not acceptable for the authors to be the sole named individuals responsible for ensuring data access.  We will update your Data Availability statement to reflect the information you provide in your cover letter. | Our human research ethics committee approval was gained with the following risk stated:  “Some of the member organisations are in countries where women's rights and human rights are vulnerable to local policies, laws and authorities.”  Our midwives’ safety in some countries could be at risk if we allow the provision of the minimum data. It is highly likely that this could leave them vulnerable. For this reason, we cannot ethically and morally provide the minimum data set. However, we have included a copy of the survey data questions. |  |
| 5. Your ethics statement should only appear in the Methods section of your manuscript. If your ethics statement is written in any section besides the Methods, please move it to the Methods section and delete it from any other section. Please ensure that your ethics statement is included in your manuscript, as the ethics statement entered into the online submission form will not be published alongside your manuscript. | The Ethics statement now is now included in the methods section L151-153. |  |
| 6. Please review your reference list to ensure that it is complete and correct. | The reference list has been reviewed. |  |

| **Reviewer #1 Comments** | **Response to Reviewer** |
| --- | --- |
| 3. Have the authors made all data underlying the findings in their manuscript fully available?  The [PLOS Data policy](https://protect-au.mimecast.com/s/mB8wCq71o3sl3LoOTp5Zaf?domain=plosone.org) requires authors to make all data underlying the findings described in their manuscript fully available without restriction, with rare exception (please refer to the Data Availability Statement in the manuscript PDF file). The data should be provided as part of the manuscript or its supporting information or deposited to a public repository. For example, in addition to summary statistics, the data points behind means, medians and variance measures should be available. If there are restrictions on publicly sharing data—e.g. participant privacy or use of data from a third party—those must be specified.  Reviewer #1: No | Our human research ethics committee approval was gained with the following risk stated:  “Some of the member organisations are in countries where women's rights and human rights are vulnerable to local policies, laws and authorities.”  Our midwives’ safety in some countries could be at risk with the provision of the minimum data and could leave them vulnerable. For this reason, we cannot ethically and morally provide the minimum data set. However, we have included a copy of the survey data questions. |
| **Reviewer Comments to the Author** | **Response** |
| Reviewer #1: Thank you for the opportunity to review this interesting paper. Below are minor editorial suggestions but overall, the manuscript is clear and well written. The research methodology sound. | Thank you for your time, Professor Sweet in reviewing this paper and your considered, thorough approach, as always. |
| Abstract: L54 Consider changing do to did – for the context of the results of this study “More than 50% of the associations reported that governments do not consult them,” | L55 ‘do’ has been replaced with ‘did’ |
| Background |  |
| L64 and thereafter WHO is spelt with a Z in Organization | Organization replaced with organisation  All other uses of the letter z in organise, organised and organisation have been replaced. |
| L81 “at greater risk of maternal death and more likely to” than what/who? Do you mean pregnant women with covid compared to non-infected or pregnant women with covid than other non-pregnant people with covid? | L82-83 This is clarified with the following statement …pregnant women or recently pregnant women with COVID-19, compared with non-pregnant women of reproductive age with COVID-19… |
| L95 and abstract you have used homebirth and home birth – please choose one and be consistent | The authors have chosen home birth and this term is now consistent throughout the paper. |
| L112-117 avoid single sentence paragraphs | These sentences have now been incorporated into the previous paragraph. L 113-115. |
| L129-133 is method not background | The sentence has now been moved to the Methods section. L144-145 |
| L148 survey responses may have been anonymous but not interviewees  L167 “Midwifery Associations could nominate that they were willing to have a follow up interview,” associations are not human – the associations representative or respondent could nominate that they were willing to have a follow up interview, | L154-158  Thank you for highlighting this inconsistency  These sentences have been moved to a new stand-alone paragraph as this relates to methods and data collection and it is stated that the interview data are not analysed or reported on in this paper. |
| L171 and elsewhere you use both SARS COV 2 and SARS-CoV-2 - – please choose one and be consistent, similarly COVID 19 and COVID-19 and COVID | The authors have edited the inconsistencies and have used SARS-COV-2 and COVID-19.  Where COVID has been used in a quotation this has been followed by [sic]. |
| L181-186 avoid single sentence paragraphs Suggest you preface the sections part in saying there were seven sections to the survey. And then put all in one paragraph | L186-190  The sentences have been amalgamated into one paragraph. |
| L188 tool or platform? | L192 Tool has been replaced with platform |
| L194 “Quantitative data were analysed using descriptive statistics” is repetitive and I don’t think needed | This has been removed |
| L209 “Survey Monkey™ platform. C” repetitive as already stated | This has been removed. |
| L213 “United Nations Population Fund (UNFPA).” Already qualified abbreviation so just use UNFPA here | At the request of the editors this has been removed from this section. |
| L215 sentence starting “This paper and represents their views and conclusions from the findings” does not make sense | L215-217  Replaced with:  This paper represents the responding ICM member Midwifery Associations views. The conclusions from the findings have been written in collaboration of the authors who participated in the conceptualisation of the study, development of the survey tool and the preparation of this manuscript. |
| L225 Table 1. Midwives’ association responses showing their geographical region (N=101) – this actually includes responders and non-responders so n should be 143 | L 226 Changed to (N=143) |
| L292 italicise first word | L293 Word now italicised |
| L299 remove brackets around 40% L319 remove brackets around 18% L320 remove brackets around 14% | Brackets removed L300, L320 and L321. |
| L330-331 “This was supported by various mainstream media reports in many countries [21]” is discussion not survey finding | Moved to discussion section L524-525. |
| L363 consider changing wording or ‘wore on’ to something like continued | L312, 364 wore on changed to continued |
| L390 add quote mark after births | L391 quote marks inserted after births |
| L405 I don’t think HIC and LMIC have been qualified | L406-407 These acronyms have now been qualified: high-income countries (HIC) and low to medium income countries (LMIC) countries |
| L445 remove brackets around 35% L454 remove brackets around 18% L459 remove brackets around 52% | Brackets removed L447, L456, L461. |
| L512 capital I in international | L515 ‘I’ now capitalised |
| L548 suggest changing COVID-19 to pandemic – to capture future events | L552: COVID-19 changed to pandemic. |

We trust that all the information you required has been provided and that our submitted documents meet your submission requirements.

We look forward to hearing from you.

Yours Sincerely

Donna Louise Hartz

PhD, Master Midwifery Studies. RN, RM, Fellow of the Australian College of Midwives

Associate Professor

University of Newcastle

AUSTRALIA
